# Borrelia burgdorferi DnaA and the Nucleoid-Associated Protein EbfC Coordinate Expression of the *dnaX-ebfC* Operon

**DOI:** 10.1128/jb.00396-22

**Published:** 2022-12-19

**Authors:** Andrew C. Krusenstjerna, Timothy C. Saylor, William K. Arnold, Jamila S. Tucker, Brian Stevenson

**Affiliations:** a Department of Microbiology, Immunology, and Molecular Genetics, University of Kentucky College of Medicine, Lexington, Kentucky, USA; b Department of Entomology, University of Kentucky College of Agriculture, Food, and Ecology, Lexington, Kentucky, USA; NCBI, NLM, National Institutes of Health

**Keywords:** *Borrelia*, DnaA, EbfC

## Abstract

Borrelia burgdorferi, the spirochete agent of Lyme disease, has evolved within a consistent infectious cycle between tick and vertebrate hosts. The transmission of the pathogen from tick to vertebrate is characterized by rapid replication and a change in the outer surface protein profile. EbfC, a highly conserved nucleoid-associated protein, binds throughout the borrelial genome, affecting expression of many genes, including the Erp outer surface proteins. In B. burgdorferi, like many other bacterial species, *ebfC* is cotranscribed with *dnaX*, an essential component of the DNA polymerase III holoenzyme, which facilitates chromosomal replication. The expression of the *dnaX-ebfC* operon is tied to the spirochete’s replication rate, but the underlying mechanism for this connection was unknown. In this work, we provide evidence that the expression of *dnaX-ebfC* is controlled by direct interactions of DnaA, the chromosomal replication initiator, and EbfC at the unusually long *dnaX-ebfC* 5′ untranslated region (UTR). Both proteins bind to the 5′ UTR DNA, with EbfC also binding to the RNA. The DNA binding of DnaA to this region was similarly impacted by ATP and ADP. *In vitro* studies characterized DnaA as an activator of *dnaX-ebfC* and EbfC as an antiactivator. We further found evidence that DnaA may regulate other genes essential for replication.

**IMPORTANCE** The dual life cycle of Borrelia burgdorferi, the causative agent of Lyme disease, is characterized by periods of rapid and slowed replication. The expression patterns of many of the spirochete’s virulence factors are impacted by these changes in replication rates. The connection between replication and virulence can be understood at the *dnaX-ebfC* operon. DnaX is an essential component of the DNA polymerase III holoenzyme, which replicates the chromosome. EbfC is a nucleoid-associated protein that regulates the infection-associated outer surface Erp proteins, as well as other transcripts. The expression of *dnaX-ebfC* is tied to replication rate, which we demonstrate is mediated by DnaA, the master chromosomal initiator protein and transcription factor, and EbfC.

## INTRODUCTION

Replication of the chromosome is a crucial yet costly process. Across all domains of life, chromosomal replication has evolved to require an initiator protein and a replicase (1, 2). For bacteria, the DNA-binding protein DnaA and the DNA polymerase III holoenzyme (DNA Pol III HE) fulfill these roles, respectively. DnaA associates at the chromosomal origin of replication (*oriC*) to high-affinity DnaA boxes and, through cooperative oligomerization to low-affinity DnaA boxes, melts the DNA to allow for loading of the helicase and the DNA Pol III HE (3–5). As expected, bacteria have developed distinct pathways to guarantee that this process is initiated appropriately for its environmental conditions.

The dual-host enzootic life cycle of Borrelia burgdorferi is intimately tied to replication. This connection is best understood at the tick-vertebrate transmission interface. In the midgut of an unfed tick, spirochetes do not replicate, but this changes when the vector takes a warm blood meal (6–8). Increased nutrient availability and temperature permit the spirochetes to replicate their genomes and alter their proteomes to permit the transmission from tick to vertebrate. For B. burgdorferi, many of these host-specific phenotypic switches are regulated by a limited repertoire of nucleic acid-binding proteins (9). A notable example is the nucleoid-associated protein EbfC, which directly regulates the production of vertebrate-stage Erp outer surface proteins and numerous other transcripts (10, 11).

EbfC is highly conserved across eubacteria, and its gene is typically the second gene in a bicistronic operon with *dnaX*, an essential component of the DNA Pol III HE (12–14). In the model organism Escherichia coli, *dnaX* codes for two subunits, the full-length Tau (τ) and shortened Gamma (γ), by translational frameshift (15–17). All bacterial replicases are composed of three domains essential to their function: a core polymerase, a sliding processivity clamp, and a clamp loading complex. Tau is the central organizer of these tripartite bacterial replicases, connecting the core polymerases on the leading and lagging strands with the processivity clamp loading complex.

For many bacterial species, DnaA functions as a transcription factor coordinating the expression of genes for various cellular pathways (18, 19). In B. burgdorferi, *dnaX-ebfC* transcript levels are tied to bacterial replication rates (20, 21), which we hypothesized could be mediated by DnaA. We found that both DnaA and EbfC contribute to the expression of the *dnaX-ebfC* operon. Additional evidence suggests that DnaA might also be involved with the production of other components of DNA Pol III HE and DNA gyrase.

## RESULTS

### DnaA binds to *dnaX-ebfC* 5′ UTR DNA.

**FIG 1:**
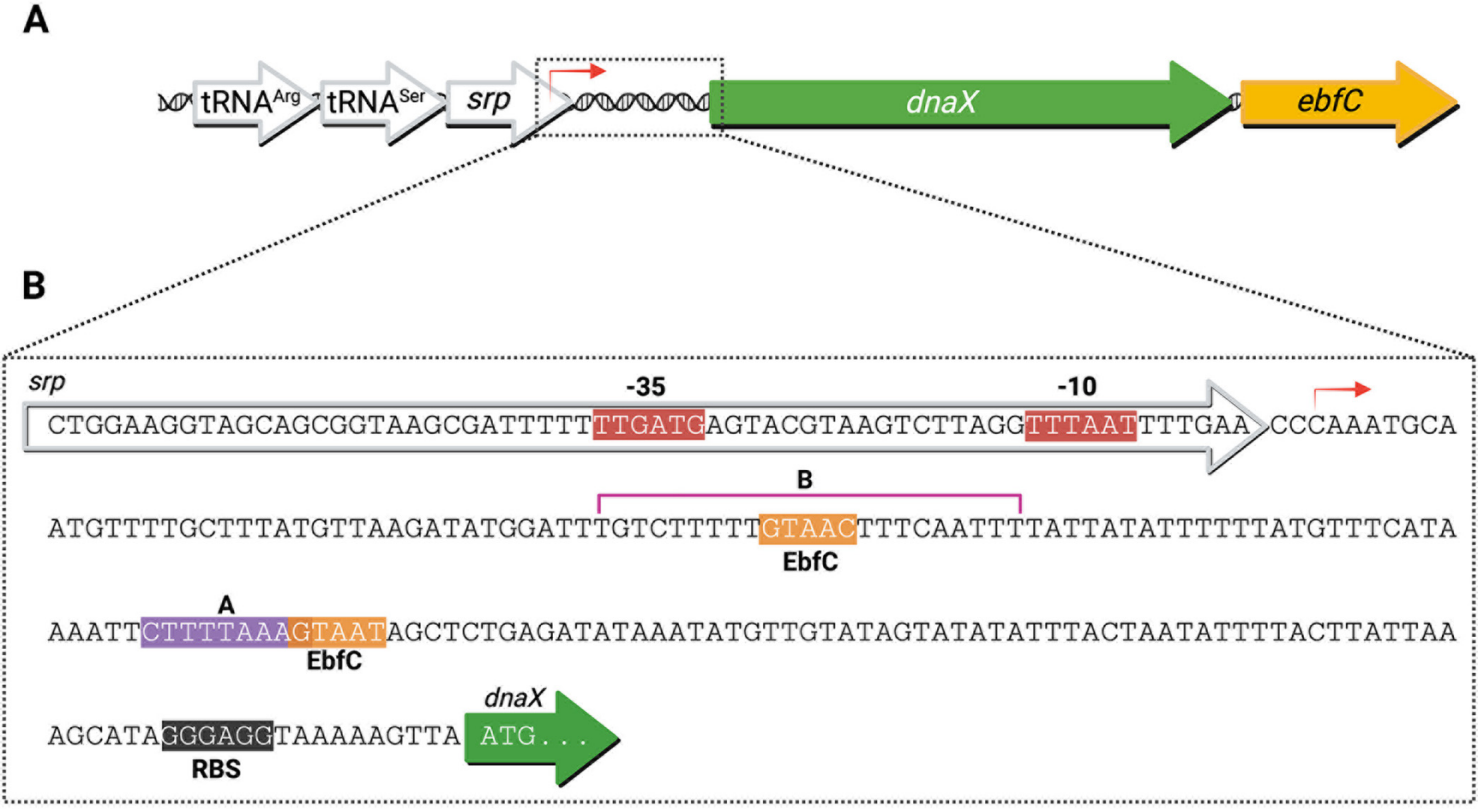
The *dnaX-ebfC* operon and its 5′ UTR. (A) The B. burgdorferi *dnaX-ebfC* locus is located downstream of genes for two tRNAs and the RNA component of the signal recognition particle (*srp*). (B) The predicted promoter (BPROM; Softberry) of the *dnaX-ebfC* operon is just within the end of the *srp* gene. The 179-nucleotide 5′ UTR contains a hypothesized DnaA box (A) and two EbfC binding motifs. The region labeled B is the sequence DnaA was also found to bind (see below).

Using promoter-reporter fusion constructs, we previously mapped the promoter for the *dnaX-ebfC* operon to be between −247 and −166 bp upstream of the *dnaX* start codon (20). This region is just within the end of the gene for the noncoding signal recognition particle RNA (*srp*) (Fig. 1A). *In silico* bacterial promoter analysis (BPROM; Softberry [22]) of this segment of DNA predicted a single σ^70^ promoter, indicating that the B. burgdorferi *dnaX-ebfC* operon has a 5′ untranslated region (UTR) of about 179 nucleotides (Fig. 1B). For context, the median 5′ UTR in B. burgdorferi is ~36 nucleotides in length (23). This significant deviation, in conjunction with the importance of this operon’s products to borrelial biology, prompted us to hypothesize that the 5′ UTR is critical to *dnaX-ebfC* regulation.

As DnaX and EbfC are evidently important for B. burgdorferi during periods of rapid replication, we first investigated whether DnaA, which is both the master replication initiator and a transcription factor in other species, can interact with *dnaX-ebfC*. Sequence-level examination of the 5′ UTR DNA of *dnaX-ebfC* revealed a potential DnaA box (Fig. 1B) (24). To test whether DnaA binds the *dnaX-ebfC* 5′ UTR DNA, we performed electrophoretic mobility shift assays (EMSAs) using a recombinant DnaA with a glutathione *S*-transferase (GST) solubility tag fused to the N terminus (GST-DnaA).

**FIG 2:**
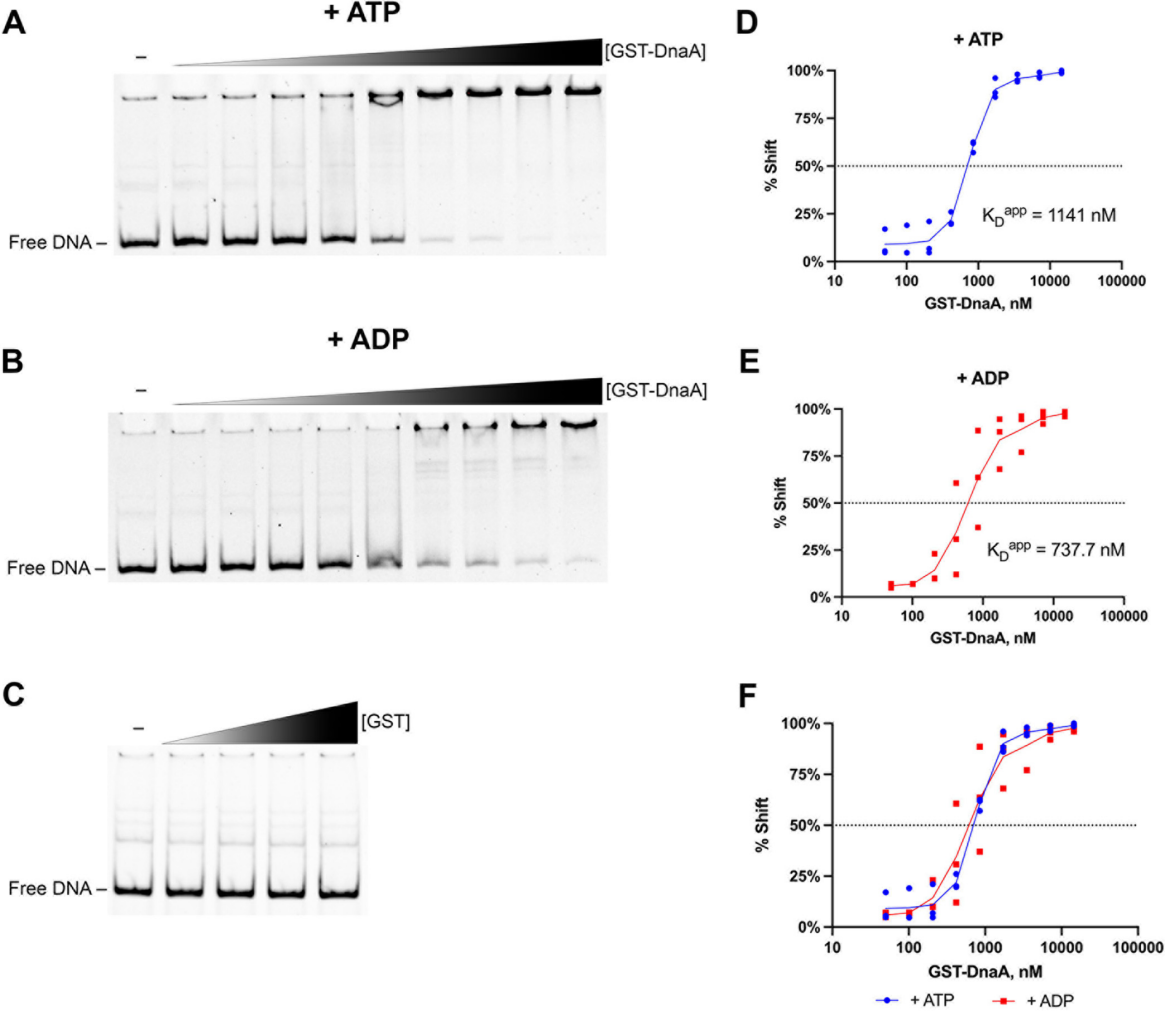
Interactions of DnaA at the *dnaX-ebfC* 5′ UTR DNA and assessment of effects of ATP and ADP. EMSAs were conducted with a fluorescent 186-mer DNA probe (100 nM) that consists of the *dnaX-ebfC* 5′ UTR. Representative EMSAs with 0.050, 0.102, 0.206, 0.419, 0.851, 1.728, 3.509, 7.126, and 14.5 μM recombinant GST-DnaA with 1 mM (A) ATP or (B) ADP. The first lanes contain the DNA probe only. (C) EMSA with 0.625, 1.25, 2.5, and 5 μM recombinant GST demonstrates that the observed GST-DnaA binding is not due to the fusion partner. (D to F) Apparent dissociation constant (*K_D_*^app^) measurements of GST-DnaA–probe interactions with ATP or ADP. Plots show the *K_D_*^app^ calculated using the densitometric data from triplicate EMSAs. The average *K_D_*^app^ is 1,141 nM for ATP reactions and 737.7 nM for ADP. The difference in GST-DnaA–probe binding between the ATP and ADP reactions was not statistically significant (*P* = 0.9835, unpaired Student’s *t* test).

In other bacterial species, the DNA binding activity of DnaA is affected by whether it has ATP or ADP bound (25–27). As our recombinant GST-DnaA was purified under mild denaturing conditions and refolded in the absence of ATP or ADP, we surmised that the protein adopted an apo- form before use. Thus, our EMSA DNA-binding reactions with GST-DnaA were supplemented with saturating concentrations of either ATP or ADP (Fig. 2A and B). We also included magnesium, a cofactor required for DnaA to bind and melt the *oriC* (28). Under these conditions, we observed concentration-dependent binding of GST-DnaA to the 186-bp *dnaX-ebfC* 5′ UTR DNA probe. Control EMSAs with purified GST confirmed that this binding was not due to the solubility tag (Fig. 2C). Quantitative analysis of densitometric data from triplicate EMSAs with GST-DnaA gave overlapping apparent dissociations constants (*K_D_*^app^) of 1,141 nM (95% confidence interval [CI], 703.5 to 1,880 nM) and 737.7 nM (95% CI, 334.8 to 1,639 nM) for ATP-DnaA and ADP-DnaA, respectively (Fig. 2D to F). These differences were not statistically significant (*P = *0.9835, unpaired Student’s *t* test). We concluded that ATP-DnaA and ADP-DnaA bind the *dnaX-ebfC* 5′ UTR DNA with similar affinities.

**FIG 3:**
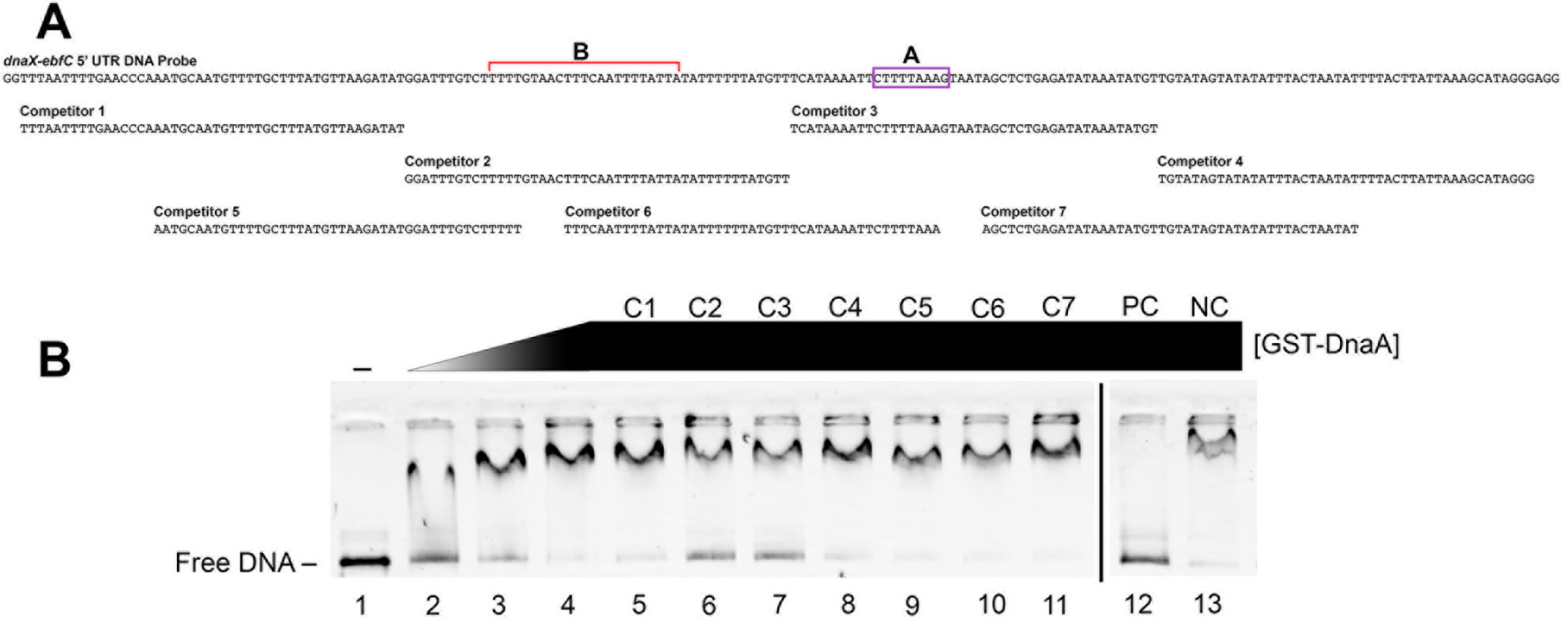
Identification of DnaA binding sites within the *dnaX-ebfC* 5′ UTR DNA. (A) Schematic of the 186-mer *dnaX-ebfC* 5′ UTR DNA probe and the seven overlapping 40-mer DNA competitors. The hypothesized DnaA box is labeled A. The region where DnaA was also found to bind is labeled B. (B) Horizontal EMSA with a polyacrylamide TBE gel using 100 nM DNA probe, GST-DnaA, ATP, and different DNA competitors. Lane 1, probe only; lanes 2 to 4, probe with 1.125, 2.25, and 4.5 μM GST-DnaA; lanes 5 to 11, probe with 4.5 μM GST-DnaA and 10 M excess of competitor DNAs (C1 to C7); lane 12, probe with 4.5 μM GST-DnaA and 10 M excess of DNA amplified from pCR2.1 containing the *dnaX-ebfC* 5′ UTR (positive control [PC]); lane 13, probe with 4.5 μM GST-DnaA and 10 M excess of DNA amplified from the empty pCR2.1 (negative control [NC]). The asterisk marks the probe-protein complex. The black line indicates the removal of extraneous lanes from the same gel.

To narrow down where DnaA binds in the *dnaX-ebfC* 5′ UTR DNA, a competition EMSA with unlabeled competitor DNAs was conducted. The DNA competitors were seven overlapping unlabeled 40-mer sequences from the labeled probe (Fig. 3A). The addition of a 10× molar excess of competitors 2 and 3 (C2 and C3) relative to the probe resulted in an appreciable increase in free DNA (Fig. 3B, lanes 6 and 7, respectively), indicating preferential binding of the protein to these regions. Competitor 3 contains the hypothesized DnaA box, but C2 does not contain a sequence resembling any previously characterized DnaA box. The inability of the two DNAs that overlap competitor 2 (C5 and C6) to diminish the shifted complex indicates that DnaA bound to a region we term region B (Fig. 3A). As a positive control, a specific DNA competitor derived from the pCR2.1 TA clone containing the *dnaX-ebfC* 5′ UTR was used. The addition of this DNA resulted in the abrogation of the protein-DNA probe complex (Fig. 3B, PC). A negative control, the nonspecific DNA derived from the empty pCR2.1 vector, did not affect the protein-probe complex (Fig. 3B, NC). These results demonstrate that DnaA binds specifically to regions A and B within the *dnaX-ebfC* 5′ UTR DNA.

### EbfC binds to the *dnaX-ebfC* 5′ UTR DNA and RNA.

**FIG 4:**
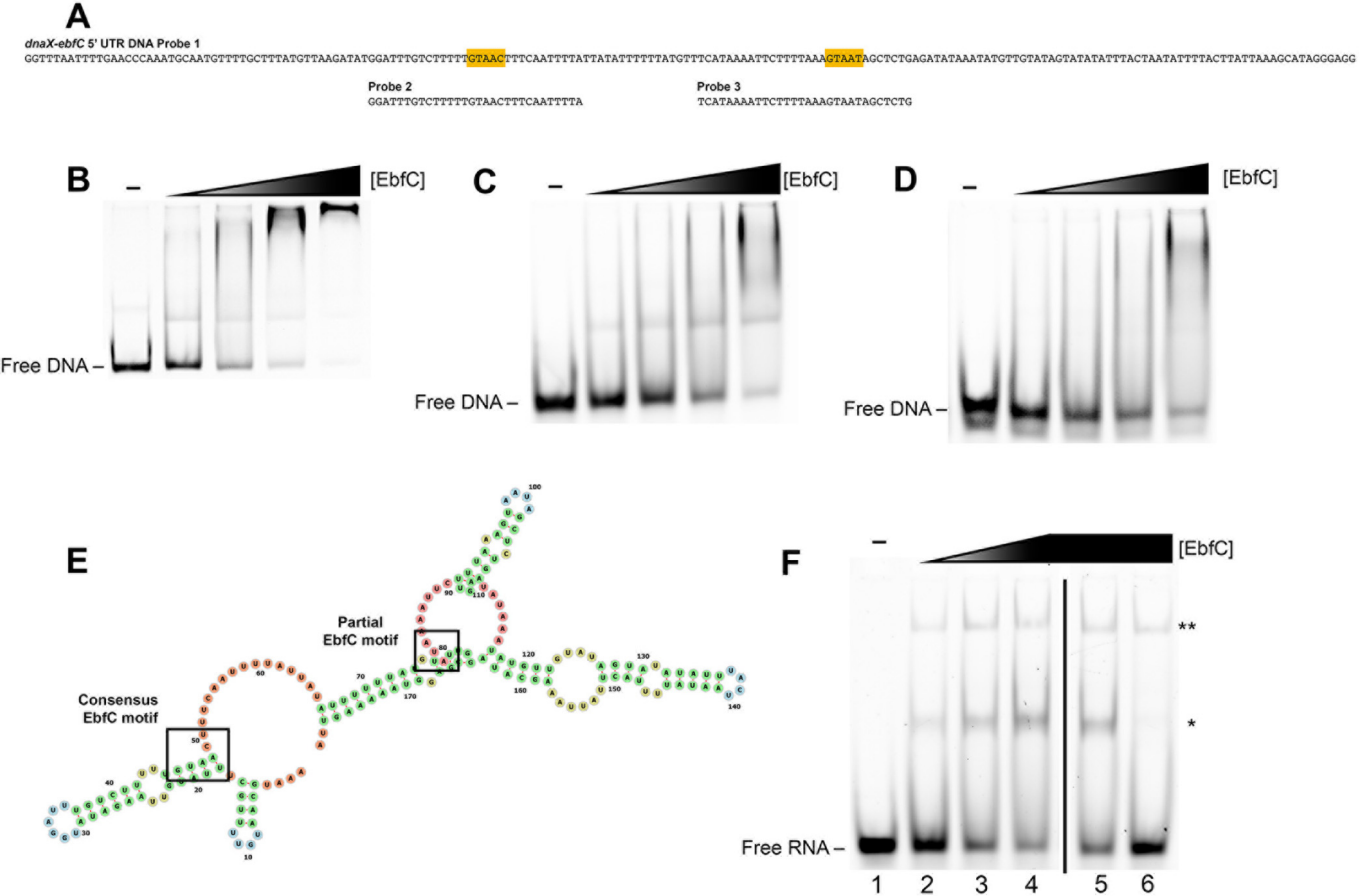
Interactions of EbfC with the *dnaX-ebfC* 5′ UTR DNA and RNA. (A to C) EMSAs with 100 nM labeled DNA probes, 2.5 ng/μL poly(dI-dC), and 3.75, 7.5, 15, or 30 μM recombinant EbfC. Lane 1 for each panel is probe only. (A) The 186-mer DNA probe from the *dnaX-ebfC* 5′ UTR. (B) The 30-mer DNA probe from the *dnaX-ebfC* 5′ UTR with the consensus EbfC motif. (C) The 30-mer DNA probe from the *dnaX-ebfC* 5′ UTR with the partial EbfC motif. (D) Predicted RNA fold structure of the 179-nucleotide 5′ UTR of *dnaX-ebfC* (RNAfold [http://rna.tbi.univie.ac.at/cgi-bin/RNAWebSuite/RNAfold.cgi]). The consensus EbfC binding motif is predicted to be part of a stem-loop, and the partial EbfC binding motif is within a knot. (E) EMSA with 20 nM EbfC, a 30-mer Alexa Fluor 647 RNA probe containing the consensus EbfC binding motif from the *dnaX-ebfC* 5′ UTR, and recombinant EbfC. Lane 1, probe only; lanes 2 to 4, probe with 5, 10, and 15 μm EbfC; lane 5, probe with 1 μM competitor *flaB* RNA and 15 μM EbfC; lane 6, probe with 1 μM competitor *dnaX-ebfC* RNA. *, lower-order complex; ** higher-order complex. All lanes are from the same gel, with the black line indicating the removal of extraneous lanes.

In addition to the DnaA boxes identified upstream of the *dnaX* open reading frame (ORF), we also noted two EbfC binding motifs (Fig. 4A). EbfC binds with highest affinity to the broken palindromic sequence GTnAC and a lesser affinity to the partial sequence GTnAT (29). EMSAs with recombinant EbfC, nonspecific poly(dI-dC) competitor, and the 186-mer DNA probe showed specific binding of the protein to this region (Fig. 4B). Further EMSAs with 30-mer DNA probes containing either the consensus or partial EbfC (Fig. 4A, probes 2 and 3, respectively) from the *dnaX-ebfC* 5′ UTR verified that the EbfC bound each of those sequences (Fig. 4C and D). The binding to the complete motif probe was appreciably more prominent than that to the partial motif probe, consistent with previous characterizations. These results demonstrate that EbfC binds two sites within the *dnaX-ebfC* 5′ UTR DNA.

During our studies, colleagues shared that EbfC could be pulled down with total RNA from extracts of B. burgdorferi (T. Van Gundy and M. Lybecker, personal communication). We hypothesized that EbfC might also bind the extended *dnaX-ebfC* 5′ UTR RNA (Fig. 4E). To examine this, we performed EMSAs with a 30-mer RNA probe containing the consensus EbfC motif from the *dnaX-ebfC* 5′ UTR. Reactions with the RNA probe and recombinant EbfC resulted in the formation of two RNA-protein complexes (Fig. 4F, lanes 2 to 4). The addition of a 50× molar excess of RNA from the constitutively expressed *flaB* did not compete away the complexes (Fig. 4F, lane 5). At the same molar excess, the unlabeled *dnaX-ebfC* RNA successfully eliminated the smaller complex (Fig. 4F, lane 6). These results demonstrated that EbfC can bind untranslated RNA 5′ of the *dnaX-ebfC* open reading frames.

### Assessment of simultaneous binding of DnaA and EbfC to the *dnaX-ebfC* 5′ UTR DNA.

**FIG 5:**
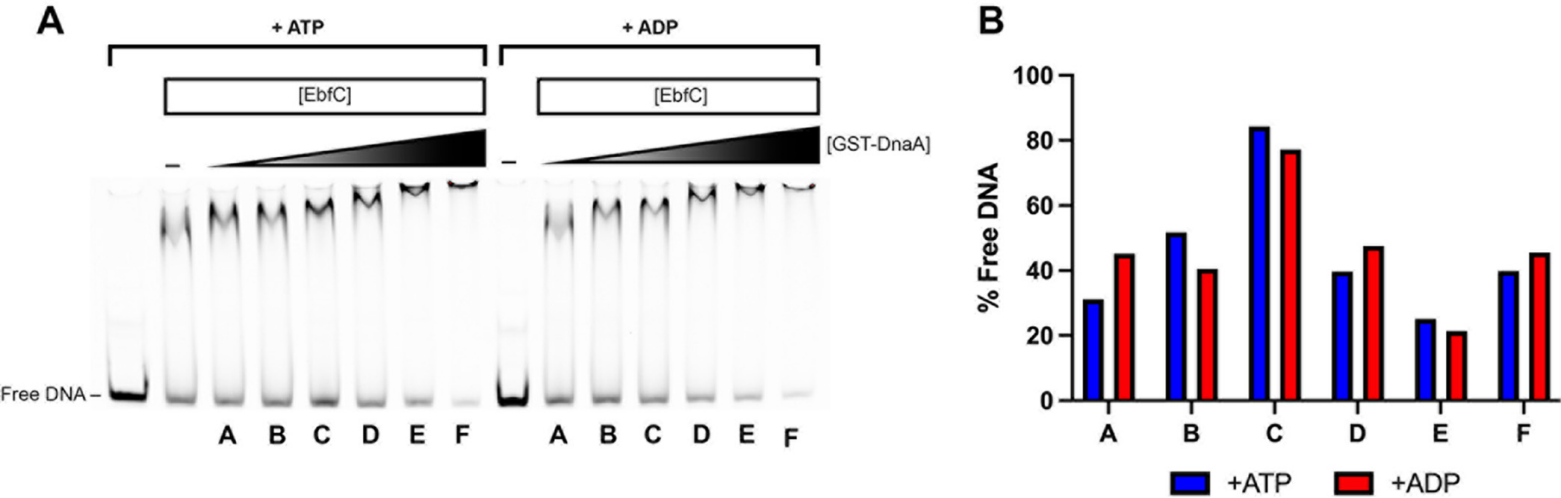
Interaction of DnaA and EbfC at the *dnaX-ebfC* 5′ UTR DNA. (A) EMSA using the *dnaX-ebfC* probe with a constant EbfC concentration of 10 μM and 0.078, 0.156, 0.312, 0.625, 1.25, or 2.50 μM GST-DnaA. Reaction A used EbfC only with the probe. Reactions B to G have an increasing concentration of GST-DnaA and constant EbfC concentration. The first eight lanes include 1 mM ATP, and the last seven include 1 mM ADP. (B) Percent free DNA for each reaction as determined by densitometry. For both ATP and ADP conditions, free DNA increased (reaction D) at lower concentrations of added GST-DnaA. At the highest GST-DnaA concentrations, free DNA decreased.

Having established that DnaA and EbfC bind the 5′ UTR DNA of *dnaX-ebfC*, we sought to determine how the proteins would interact in this region. To examine this, EMSA reaction mixtures with constant EbfC and increasing GST-DnaA were prepared (Fig. 5A). The constant EbfC concentration used allowed for roughly equivalent levels of bound and unbound DNA (Fig. 5A, lane A). With increasing concentrations of GST-DnaA (Fig. 5A, lanes B to G), the DNA-protein complex migrated more slowly in the gel. Densitometry showed an initial increase of free DNAs at low levels of added GST-DnaA (Fig. 5B, lanes B to D), followed by a decrease at the highest GST-DnaA concentrations (Fig. 5B, lanes E to G). This pattern appeared to be the same whether ATP-DnaA or ADP-DnaA was used. These data suggest that the two proteins may bind cooperatively to the *dnaX-ebfC* 5′ UTR DNA. The precise stoichiometry and kinetics of the DNA interactions are undoubtedly complex, given the proximity of the binding sites for each protein.

### DnaA enhances the expression of *dnaX-ebfC*, while EbfC inhibits this effect.

**FIG 6:**
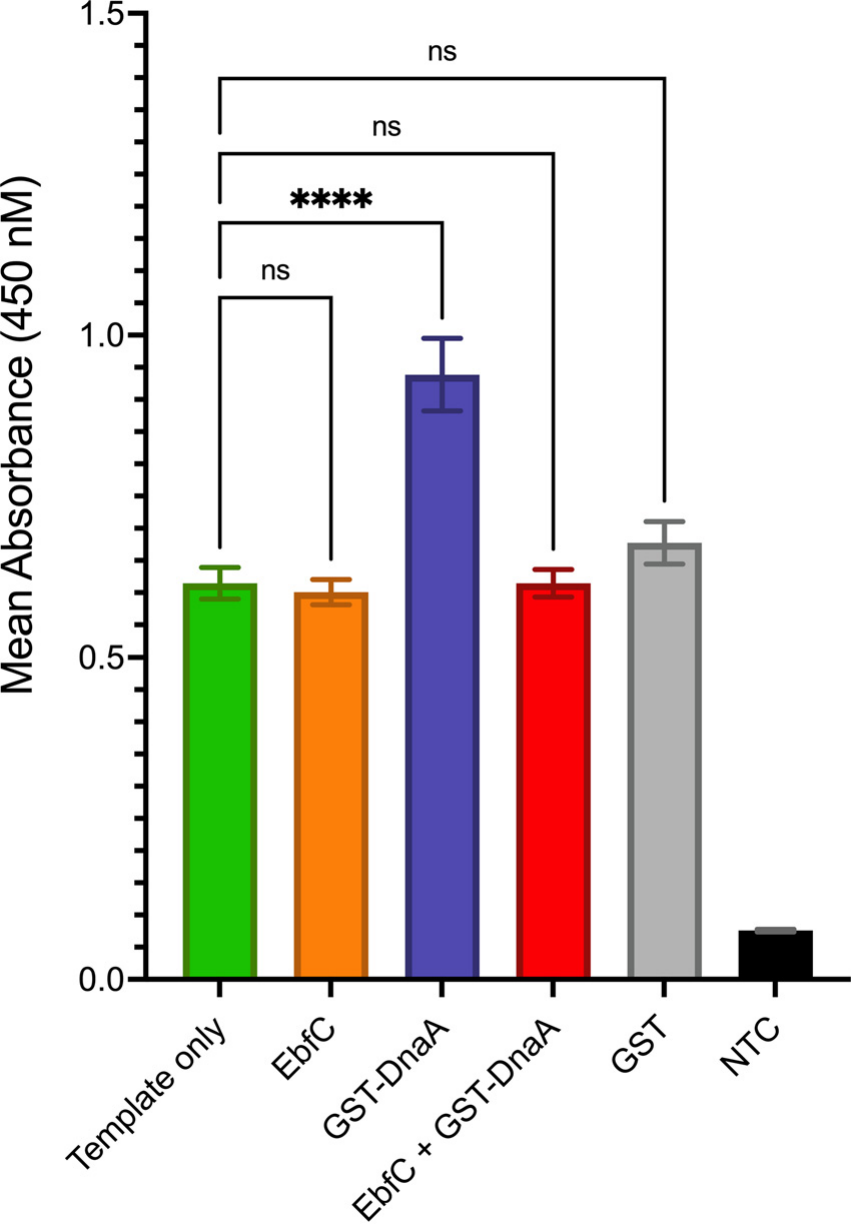
Effects of purified recombinant protein on *dnaX-ebfC in vitro* expression. Reactions used ~65 nM linear DNA containing 247 bp upstream of the *dnaX* start codon fused to *gfp*. GST-DnaA, EbfC, or GST was added at a final concentration of 1 μM. Reporter GFP levels were quantified by ELISA and are reported as mean absorbances from triplicate reactions. The error bars indicate the standard errors of the means (SEM). ****, *P* < 0.001 relative to the template-only reaction. GST served as a control to demonstrate the specific activity of the recombinant proteins. NTC, no-template control; ns, not significant.

Having established the interactions of DnaA and EbfC at the *dnaX-ebfC* DNA/RNA, the next logical step was to determine whether the proteins affect the expression of *dnaX-ebfC*. As both proteins are essential for the survival of B. burgdorferi (29), analyses with deletion mutant bacteria could not be done. Instead, cell-free *in vitro* transcription/translation assays with Escherichia coli S30 extracts were performed. This method has been a useful tool for quantifying DNA-binding proteins’ effects on borrelial gene and protein expression and has the added benefit of lacking B. burgdorferi DnaA or EbfC (10, 11, 30). The template for these reactions was a linear DNA that contains the 247 bp 5′ of *dnaX*, including the transcriptional promoter, fused to *gfp* (20). The addition of GST-DnaA to the *in vitro* reaction mixture resulted in a significant increase (*P* = 0.0004, Brown-Forsythe and Welch analysis of variance [ANOVA]) in the production of the reporter green fluorescent protein (GFP) (Fig. 6). Control reactions with added GST alone did not significantly change GFP levels.

The *in vitro* reactions with recombinant EbfC protein alone did not result in a significant change in GFP output (Fig. 6). Only in the presence of GST-DnaA did EbfC exhibit an effect, reducing reporter output to template-only levels (Fig. 6). These results indicate that *in vitro*, EbfC alone does not exert a regulatory effect, but it does inhibit the DnaA-dependent activation of *dnaX-ebfC* expression. This effect on coupled transcription and translation, combined with the apparent lack of competition between EbfC and DnaA for binding to the 5′ UTR DNA (Fig. 5), suggests that the effect of EbfC may be through its interaction with RNA.

### DnaA binds to 5′ DNA of other essential replication genes.

The canonical bacterial DNA Pol III HE of E. coli consists of nine subunits. Comparatively, the borrelial replicase, like other spirochetes, has a pared-down repertoire (Table 1). B. burgdorferi has the bare minimum required to replicate its chromosome: a polymerase (DnaE, α), processivity clamp (DnaN, β), clamp loader (DnaX, τ), and clamp opener (HolA, δ). B. burgdorferi *dnaX* does not appear to have the *cis* element for translational frameshift that produces the Gamma subunit, and so it might produce only the Tau subunit (see Fig. S1 in the supplemental material). Moreover, genes for the subunits involved with fidelity (DnaQ, ε; HolE, θ) are absent from the borrelial genome.

**TABLE 1:** Subunits of DNA polymerase III holoenzyme

Subunit	Function	Homolog in^*a*^:			
		E. coli	B. burgdorferi	Treponema pallidum	Leptospira interrogans
DnaE, α	Polymerase activity	B0184	BB0579	TP0669	LIC10222
DnaN, β	DNA clamp; locking Pol III to DNA to increase processivity	B3701	BB0438	TP0002	LIC10002
DnaQ, ε	3′→5′ exonuclease activity	B0215	—	TP0643	—
DnaX, τ and γ	Dimerization of core enzymes (τ); clamp loader (γ)	B0470	BB0461	TP1005	LIC13474
HolA, δ	Clamp opener	B0640	BB0455	TP0588	—
HolB, δ’	Clamp loader	B1099	—	—	LIC13497
HolC, χ	Interaction with SSB^*b*^	B4259	—	—	—
HolD, Ψ	Stabilizing γ and χ interaction	B4372	—	—	—
HolE, θ	Proofreading activity	B1842	—	—	—

a —, the organism lacks an identifiable homolog.

b SSB, single-standed DNA-binding protein.

Since we found that DnaA regulates *dnaX-ebfC*, we posited that DnaA might also be involved in regulating the other subunits of the replicase and itself. With this aim in mind, the 5′ intergenic regions of *dnaN*, *dnaE*, *holA*, and *dnaA* were used as labeled DNA probes for EMSA analysis.

**FIG 7:**
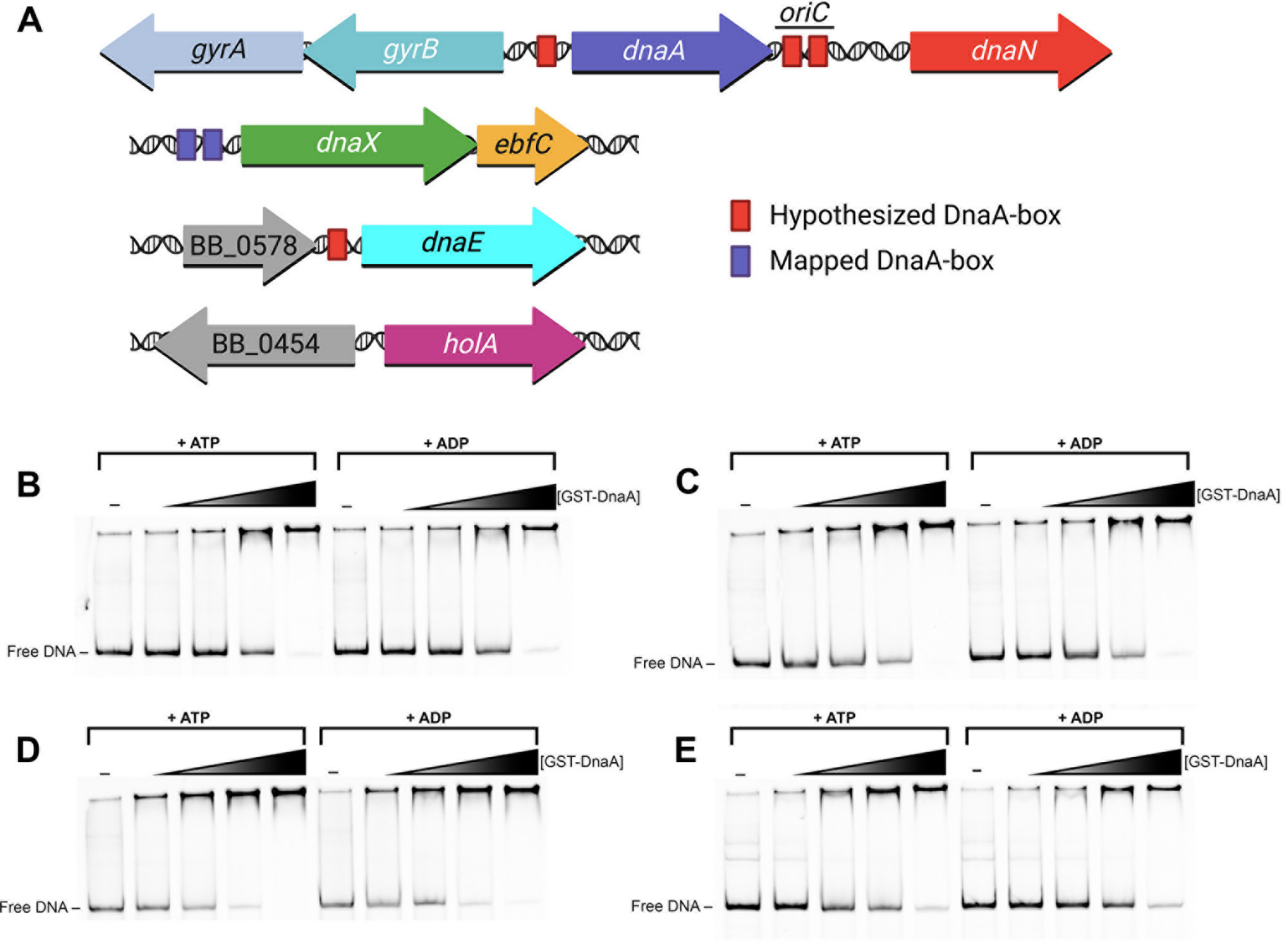
EMSAs with PCR amplified probes containing the intergenic regions 5′ of *dnaA* and the DNA pol III HE genes. (A) Schematic of the loci subject to EMSA analysis. Red rectangles indicate predicted DnaA boxes. Purple rectangles indicate mapped DnaA boxes. Each EMSA reaction (B to E) used 100 nM probe, 2.5 ng/μL poly(dI-dC), and either 0.56, 1.125, 2.25, or 4.5 μM GST-DnaA. The first lane for each EMSA is probe only. The first five lanes of each panel included 1 mM ATP, and the last five included 1 mM ADP. (B) Probe containing the *oriC*–*dnaA-dnaN* intergenic region. (C) Probe containing the intergenic region of *gyrB-dnaA*. (D) Probe containing the intergenic region 5′ of *dnaE*. (E) Probe containing the intergenic region 5′ of *holA*.

The *dnaN* gene (BB_0438) codes for the processivity clamp (β) of the DNA Pol III HE and is located downstream of the *dnaA* gene (BB_0437) (Fig. 7A). The B. burgdorferi *oriC* is between *dnaA* and *dnaN* and contains two hypothesized DnaA boxes (31). EMSAs demonstrated the binding of recombinant DnaA to a labeled probe that consisted of the 240-bp *dnaA-dnaN*–*oriC* region (Fig. 7B). The free probe was almost entirely bound by the higher levels of DnaA. There were no appreciable differences in ATP- or ADP-DnaA binding to the *oriC* probe. These results are consistent with the predicted functions of DnaA at *oriC* to control replication initiation. Further, the placement of the *oriC* in proximity to the *dnaN* promoter suggests that DnaA may regulate this gene, as it does in the firmicute Bacillus subtilis (32).

We also found that DnaA bound the 186-bp 5′ intergenic region of *dnaA* and the opposite-facing *gyrB* (BB_0436) (Fig. 7C), a location that contains another hypothetical DnaA box (Fig. 7A) (31). The *gyrB* gene is located upstream of *gyrA* and presumably forms an operon (*gyrBA*), as in other prokaryotes. The *gyrBA* genes code for subunits A and B of the DNA gyrase, a topoisomerase essential in bacterial replication. For the *gyrB-dnaA* region, the free probe was almost entirely bound at the highest protein concentrations. ATP-DnaA and ADP-DnaA bound similarly to this DNA. These results show that DnaA strongly binds to its promoter region, suggesting that it may undergo autoregulation as in other bacteria (25, 32–36).

The gene for the DNA polymerase DnaE (α, BB_0579) is located downstream of the gene for a methyl-accepting chemotaxis protein (*mcp-1*, BB_0578) (Fig. 7A). The intergenic region between these genes is 130 bp, and it contains a potential DnaA box (24). EMSAs with recombinant DnaA showed binding to the *dnaE* probe (Fig. 7D). DnaA with either ATP or ADP again bound the DNA similarly.

The *holA* gene (δ, BB_0455) promoter overlaps with a neighboring upstream gene (BB_0454) that codes for a predicted lipid galactosyltransferase (Fig. 7A). This intergenic region is small, consisting of only 55 bp, and does not contain a sequence that resembles any known DnaA box. Despite this, EMSAs showed that ATP- and ADP-DnaA bound to the 5′ *holA* DNA (Fig. 7E).

**FIG 8:**
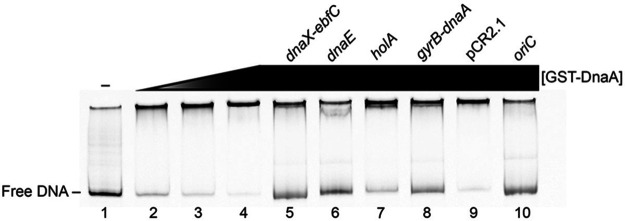
Relative affinities of DnaA for labeled *oriC* probe in comparison with other loci. Competitive EMSA using a final labeled *oriC* probe concentration of 100 nM for each lane. Lanes 2 to 4 have concentrations of 1.125, 2.25, and 4.5 μM GST-DnaA, respectively, and lanes 5 to 10 have constant levels of 4.5 μM GST-DnaA. Competitor DNAs were amplified using unlabeled M13 forward and reverse primers from TA constructs and added to reaction mixtures at a 10× molar excess relative to the *oriC* probe (lanes 6 to 10).

To compare the relative affinities of DnaA for all these regions, a competition EMSA was performed using the labeled *oriC* DNA as the probe. This DNA was selected because it is the primary site for DnaA interaction and is thus an ideal standard. At a 10× molar excess, the unlabeled DNA competitors from the *dnaX-ebfC* 5′ UTR, *dnaE*, and *gyrB*-*dnaA* considerably depleted the DnaA-*oriC* complex (Fig. 8, lanes 5, 6, and 8, respectively). The competitor DNA from *holA* partially diminished the observed shift (Fig. 8, lane 7), consistent with the data above (Fig. 7E). The negative-control DNA derived from the empty pCR2.1 vector, used for all DNAs, failed to compete (Fig. 8, lane 9). The unlabeled *oriC* DNA successfully depleted the DnaA-*oriC* probe complex (Fig. 8, lane 10).

**FIG 9:**
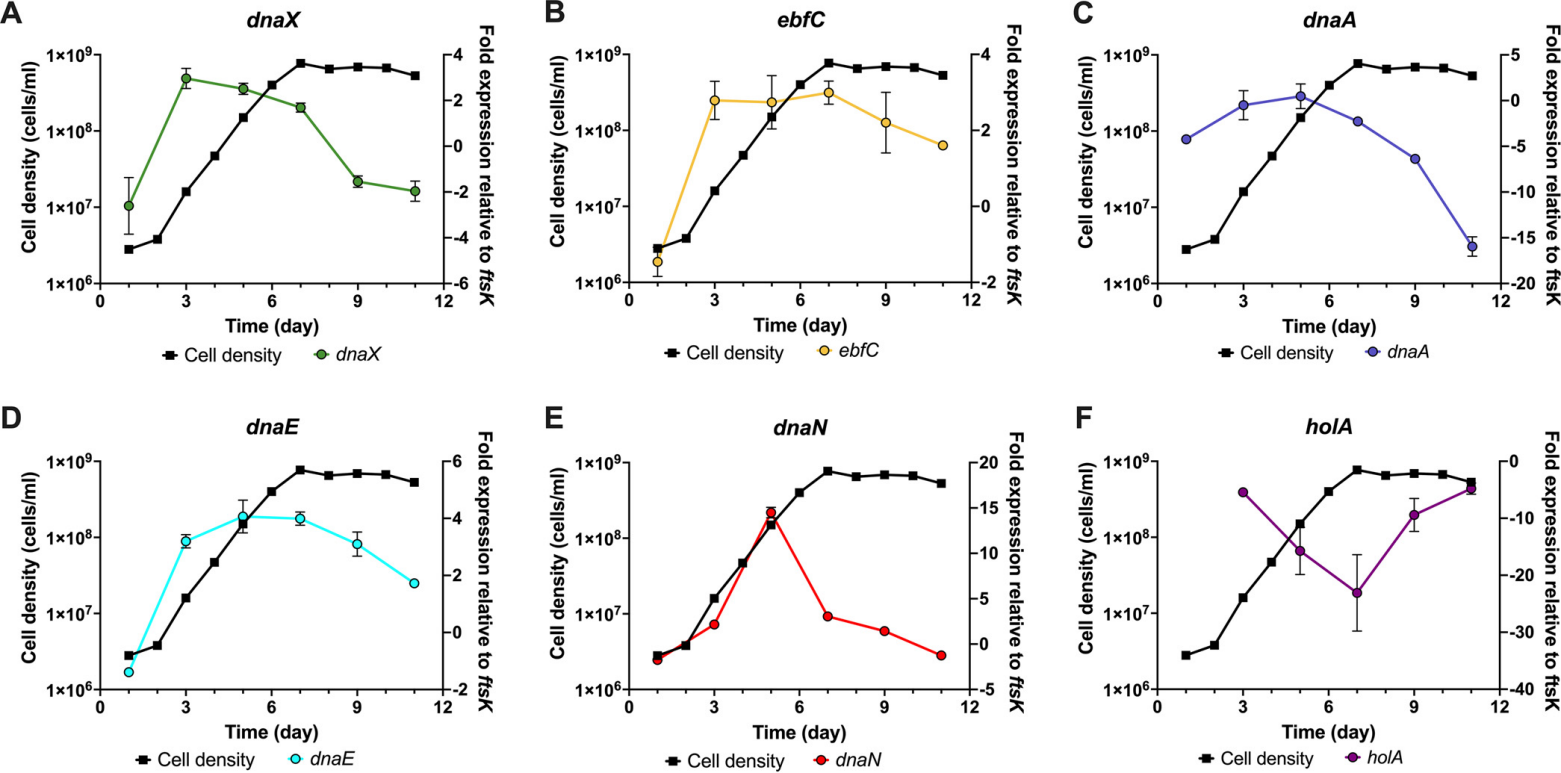
Tracking DNA Pol III HE gene expression changes during growth phases. The cell density of B. burgdorferi (left *y* axes) was measured every 24 h, and the relative transcript levels (right *y* axes) were analyzed every 48 h. The fold expression of transcript was calculated by normalization to the constitutive *ftsK* gene and reported as the average for triplicate reactions. The error bars indicate the SEM.

With knowledge of the physical interactions of DnaA at these loci, we next sought to track the transcriptional dynamics of these replication genes during the different growth phases of cultured B. burgdorferi. As we have established DnaA as an activator of *dnaX-ebfC* transcription *in vitro*, we hypothesized that they would have similar expression profiles. Indeed, the data show that the relative levels of *dnaX* and *ebfC* transcripts rose with *dnaA* as the culture entered the exponential phase (Fig. 9A to C). Moreover, *dnaX* and *dnaA* declined as the spirochetes entered the stationary growth phase, while *ebfC* remained stable for some time but eventually decreased. This divergence is consistent with our previous finding that *ebfC* has another promoter within the *dnaX* open reading frame (20).

The transcript levels of *dnaN* and *dnaE* also followed the pattern seen with *dnaA* (Fig. 9D and E). The relative expression level for both genes peaked at mid-exponential phase and steadily decreased into stationary phase. The *holA* gene, in contrast, exhibited a unique expression profile compared to the other components of the DNA Pol III HE. While the other genes’ transcripts decreased during stationary phase, *holA* transcript levels increased (Fig. 9F). Furthermore, the relative abundance of the *holA* transcripts were considerably lower than those of other DNA polymerase III subunit mRNAs.

## DISCUSSION

Tight and coordinated regulation of chromosomal replication is a key biological process. The evolution of such regulatory networks was significant for unicellular organisms, which can inhabit dynamic environments that challenge cellular homeostasis. B. burgdorferi has evolved the need to concomitantly modulate DNA replication and gene expression during its consistent cycling between tick vectors and vertebrate hosts. These requirements can be appreciated at the *dnaX-ebfC* locus, where DnaX (Tau) assembles with the DNA Pol III HE to replicate the chromosome, while EbfC binds at sites throughout the nucleoid and presumably affects chromatin structure. Previously, we reported that the expression of *dnaX-ebfC* depends on the replication rate (20, 21), but the question of how remained unanswered. This work indicates that this connection can be explained by direct interactions of DnaA and EbfC at the 5′ UTR of *dnaX-ebfC*.

DnaA is well established to be a transcription factor in other bacterial species (19). For B. burgdorferi, our data show that DnaA binds 5′ of the *dnaX-ebfC* operon to enhance gene expression. This is a logical pathway to evolve, given that DnaA and DnaX are directly linked to chromosomal replication. Moreover, this mechanism is perhaps conserved, as two DnaA boxes are located upstream of the E. coli *dnaX-ebfC* operon (37).

**FIG 10:**
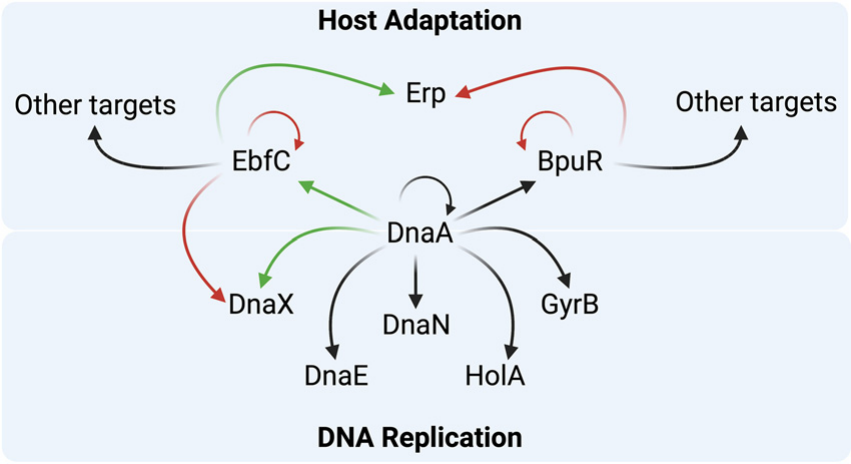
Diagram of experimentally confirmed and hypothesized effects of DnaA on B. burgdorferi protein expression. Green arrows indicate positive effects, red arrows indicate negative effects, and black arrows indicate predicted effects. DnaA positively influences expression of both DnaX and EbfC. EbfC counteracts the effect of DnaA on the *dnaX-ebfC* operon, acts as an antirepressor to stimulate transcription of *erp* operons, and also affects expression of numerous other transcripts. DnaA binds over the *bpuR* promoter and is hypothesized to repress transcription of that gene, while BpuR has been demonstrated to repress its own translation, enhance repression of *erp* operons, and affect expression of several other borrelial proteins. DnaA also binds to its own promoter region, as well as those of *dnaE*, *dnaN*, *gyrB*, and *holA*, and is hypothesized to affect expression of those operons.

Transcriptional control of bacterial DNA replication machinery through DnaA has also been observed in Caulobacter crescentus. A Gram-negative aquatic bacterium, C. crescentus exhibits a complex cell cycle defined by asymmetric division into stalked and swarmer progeny, where DNA replication is initiated only once per cell cycle (38). In C. crescentus, DnaA controls the transcription of two DNA replication genes, the helicase gene *dnaB* and the DNA Pol III HE ε-subunit, *dnaQ* (39). There is also evidence of an unknown repressor binding to a conserved promoter motif upstream of C. crescentus *dnaX*, *dnaN*, *dnaA*, *dnaK*, and *gyrB* genes (40). Our work suggests that a similar regulation network may exist in B. burgdorferi, where DnaA binds upstream of *dnaX*, *dnaN*, *dnaE*, *holA*, *dnaA*, and *gyrB* (Fig. 10). The effect of DnaA on these borrelial genes, aside from *dnaX*, remains to be assessed.

The potential global transcriptional effects of DnaA in B. burgdorferi raise the question of what controls the master initiator. In other bacteria, the cellular ATP/ADP ratio affects the function of DnaA (25–27). Our data failed to identify a measurable difference between ATP- and ADP-DnaA binding to any tested sequence. This suggests that either B. burgdorferi DnaA lacks sensitivity to those nucleotides under the tested conditions or the protein has novel control mechanisms. The latter possibility is especially intriguing due to the lack of protein homologs such as DiaA and Hda, which in other bacteria regulate the hydrolysis of ATP bound to DnaA (41, 42). It is also possible that sequence differences in the borrelial nucleotide-binding/ATPase domain could explain our results. While Walker A and B motifs and lysine residues whose acetylation prevents nucleotide binding are conserved in the borrelial DnaA, one alanine is not (43, 44) (Fig. S2). In E. coli, mutation of this residue (A184 to V) results in reduced DnaA affinity for ATP (45). Interestingly, B. burgdorferi DnaA has a serine at this position. Whether this residue or other factors account for our results remains to be examined.

The idea of novel DnaA-DNA interactions in B. burgdorferi has previously been hypothesized, since the borrelial homolog differs in three of eight amino acid residues in the DNA-binding C terminus that are conserved in other species (46). In the E. coli DnaA, two of the divergent amino acids, T451 and S453, form base-specific hydrogen bonds with DNA (47). This suggests that the DnaA protein of B. burgdorferi may recognize unique DNA sequences. Our data showing DnaA binding 5′ of *holA* and *dnaX-ebfC* 5′ UTR B, which lack sequence similarity to the characterized DnaA boxes of other species, points to this possibility. Investigation of genome-wide distribution of bound DnaA in B. burgdorferi to identify the borrelial DnaA box sequence is under way.

In addition to the regulatory activity of DnaA, our studies demonstrated that EbfC affects *dnaX-ebfC* expression. That is consistent with previous studies by our group, which found that overexpression of EbfC resulted in a decrease of *dnaX* transcript (20). The present work demonstrates that EbfC regulates *dnaX-ebfC* expression by counteracting DnaA-dependent transcriptional activation. The lack of competition between DnaA and EbfC in binding to the *dnaX-ebfC* 5′ UTR DNA suggests that EbfC may counteract the effect of DnaA by binding to *dnaX-ebfC* mRNA, which could alter secondary structure and affect transcription or translation. Further investigations to tease apart the mechanism of regulation are complicated by the fact that mutagenesis of the *dnaX-ebfC* 5′ UTR DNA will affect the structure of the mRNA.

The fluctuations from tick and vertebrate hosts have been a constant for B. burgdorferi for thousands of years. These divergent biological environments have forced the spirochete to evolve a regulatory network to adapt and endure appropriately. In this study, we provided evidence that B. burgdorferi uses its DnaA protein to promote the expression of an operon crucial to its replication and survival. DnaX, the central organizer of the DNA Pol III HE, is essential for chromosomal replication. EbfC affects expression of *dnaX-ebfC*, the genes for vertebrate-stage outer surface Erp proteins, and many other borrelial genes. DnaA activity at *dnaX-ebfC* thus permits coordinated expression of replication machinery and a protein needed for borrelial host infection (Fig. 10). B. burgdorferi DnaA also binds over the promoter of *bpuR*, an RNA- and DNA-binding protein that, in turn, affects expression levels of Erp and several other borrelial proteins (24). We hypothesize that B. burgdorferi DnaA also regulates the production of other proteins involved in replication and infection processes.

## MATERIALS AND METHODS

### Bacterial strains and culture conditions.

All studies utilized derivatives of the B. burgdorferi type strain, B31, and were cultivated in liquid Barbour-Stoenner-Kelly II (BSK-II) medium (48). For RNA analyses, cultured bacteria were subject to a temperature shift wherein spirochetes were grown at 23°C to mid-exponential phase (~5 × 10^7^ cells/mL), passaged 1:100 into fresh BSK-II medium, and incubated at 34°C. Stationary-phase bacteria were collected from cultures at or above 10^8^ cells/mL. Culture cell densities were determined by direct counting in a Petroff-Hausser counting chamber with dark-field microscopy.

### Recombinant proteins.

EbfC was cloned into pET200 and expressed with an N-terminal 6×His affinity tag. Recombinant EbfC was expressed in Escherichia coli Rosetta II cells and purified using MagneHis Particles (Promega) as previously described (24, 49).

DnaA was cloned into pET-21a(+) with an N-terminal glutathione *S*-transferase (GST) moiety by the University of Kentucky Protein Core. A GST tag was selected to enhance the solubility of DnaA, which in other bacteria is known to aggregate into insoluble inclusion bodies (50, 51). Recombinant GST-DnaA protein was expressed in E. coli BL21(DE3) cells with 1 mM isopropyl-β-d-thiogalactopyranoside (IPTG) overnight at room temperature. Recombinant GST was expressed in E. coli Rosetta II cells containing pGEX-3x. Cells were collected by centrifugation and frozen at −80°C.

Frozen GST-DnaA-expressing cells were resuspended in ice-cold washing/binding buffer (4.2 mM Na_2_HPO_4_, 2 mM KH_2_PO_4_, 140 mM NaCl, 10 mM KCl) and then supplemented with 5% (vol/vol) B-PER bacterial protein extraction reagent (Thermo Scientific) before sonication. The soluble fraction was separated by centrifugation and discarded. The insoluble fraction was retained and processed using mild solubilization per Qi and colleagues (52). Briefly, the insoluble pellet was washed three times with inclusion body wash buffer (20 mM Tris, 300 mM NaCl, 1 mM EDTA, 1% Triton X-100, 1 M urea, pH 8.0) followed by two washes with phosphate-buffered saline (PBS), pH 7.4, to remove detergent. The final pellet was resuspended in PBS with 2 M urea and frozen at −20°C for at least 12 h. The frozen suspension was then thawed at room temperature and dialyzed at 4°C for at least 3 h in 1 L of PBS with 1 M urea, 0.5 M urea, and no urea. The clarified soluble protein suspension was purified using MagneGST glutathione particles (Promega) according to manufacturer recommendations. Recombinant GST was purified using the same MagneGST conditions but under standard soluble conditions.

Purified proteins were dialyzed against EMSA binding buffer (50 mM Tris-HCl, 50 mM KCl, 1 mM EDTA, 1 mM phenylmethanesulfonyl fluoride [PMSF], 1 mM dithiothreitol [DTT], 0.01% Tween 20, 10% glycerol) overnight at 4°C and concentrated with 10-kDa-molecular-weight-cutoff (MWCO) Amicon centrifugal filters (Sigma). Protein concentrations were determined by the Bradford assay, and purity was assessed by SDS-PAGE with Coomassie brilliant blue staining. Final protein preparations were stored at −80°C in 12-μL working aliquots.

### TOPO plasmid constructs.

For these studies, DNA regions of interest were PCR amplified from B. burgdorferi B31 genomic DNA with *Taq* polymerase and TA cloned into pCR2.1 (Thermo Fisher). Constructs were sequenced to guarantee sequence fidelity.

### EMSAs.

Labeled and unlabeled nucleic acids used are described in Table S1. Oligonucleotides for these assays were synthesized by Integrated DNA Technologies (IDT, Coralville, IA). For use as EMSA probes, DNAs were conjugated with a 5′ IRDye800 fluorescent tag, while RNAs had a 5′ Alexa Fluor 647 tag. Short DNA probes (<60 bp) were produced by heating two complementary oligonucleotides to 95°C for 5 min and gradually cooling them to room temperature. Long DNAs (>60 bp) were produced by PCR amplification from TA cloned plasmids using forward and reverse M13 primers, with a fluor tag on the 5′ end of M13 Reverse. PCR products were treated with exonuclease I (New England Biolabs) to remove residual primers. The reaction products were precipitated by adding a 1/10 volume of 3 M sodium acetate and a 2.5× volume of ice-cold 100% ethanol. Glycogen was added at a final concentration of 1 mg/mL to aid pellet visualization. DNA was pelleted by centrifugation at 4°C and washed twice with ice-cold 70% ethanol to remove excess salt. The final DNA pellet was air dried at room temperature for 15 min and resuspended in 50 μL of TE buffer. Nucleic acid concentrations were determined spectrophotometrically and diluted to 1 μM. Final DNA probes were stored at −20°C in working doses of 12 μL.

EMSAs were performed essentially as described previously (11, 53). Briefly, protein-nucleic acid binding reactions were carried out at room temperature for 15 min in EMSA binding buffer using either 100 nM or 20 nM probe. All reaction mixtures with GST-DnaA were supplemented with 1 mM MgCl_2_ and 1 mM either ATP or ADP and incubated on ice for 15 min before addition of DNA. When appropriate, the nonspecific competitor poly(dI-dC) (Roche) was added to each reaction mixture before the probe at a final concentration of 2.5 ng/μL (54). Competitor nucleic acids were also added to reactions before the labeled probe. Prior to electrophoresis, 6× EMSA loading dye (0.8 mg/mL orange G, 15 mg/mL Ficoll 400) was added to each reaction mixture. Electrophoresis was performed with 6% or 10% Novex Tris-borate-EDTA (TBE) gels (Thermo Fisher) or in-house 6% polyacrylamide TBE gels. The in-house polyacrylamide TBE gels (40 mL) for horizontal electrophoresis were produced by combining 6 mL 40% acryl/bis 29:1 (VWR), 4 mL of 5× TBE (pH 7.5) (445 mM Tris, 445 mM boric acid, 5 mM EDTA), 30 mL double-distilled water (ddH_2_O), 300 μL 10% APS (Fisher), and 100 μL of TEMED (National Diagnostic). The gels were prerun in 0.5× TBE buffer at 12.5 V/cm for 30 min. Samples were loaded and resolved at 12.5 V/cm until the dye front reached the bottom of the gel.

The apparent *K_D_* of GST-DnaA for the *dnaX-ebfC* 5′ UTR DNA probe was determined by densitometry from triplicate EMSAs using ImageLab software (Bio-Rad). Lanes were manually set to the EMSA gel. The free DNA and shifted bands were manually selected, and the intensities were reported as lane percent. These values were subsequently plotted against the corresponding protein concentrations. The plotted data were then subjected to nonlinear regression analysis using Prism 9 software. An unpaired *t* test was performed to assess statistically significant differences (α = 0.5) between the ATP and ADP data. Since the method for purifying DnaA protein involved denaturation and then renaturation, it is possible that a proportion of protein remained denatured and that binding affinities for native protein could be greater than calculated. However, all comparative binding analyses were performed with the same batches of purified DnaA, so relative *K_D_* values with ATP or ADP would have the same proportions.

### Coupled *in vitro* transcription/translation assays.

Coupled *in vitro* transcription/translation assays were performed as previously described (10, 11, 30). A linear DNA template was amplified from pAAB206 using M13 forward and reverse primers (20). This amplicon contains 247 bp 5′ of *dnaX-ebfC* fused to *gfp*. Experiments were performed using the E. coli S30 extract system for linear templates (Promega). The 50-μL reaction mixtures were composed of kit components, 2,100 ng of template DNA (~65 nM), 40 U of RiboGuard RNase inhibitor (Lucigen), and a 1 μM concentration of the proteins of interest, when appropriate. Purified GST was used as a control. Reaction mixtures were incubated at 37°C for 2 h and promptly iced for 5 min to stop transcription/translation. Each reaction set was performed in triplicate.

The *in vitro* protein products were precipitated by adding 4 volumes of ice-cold acetone and incubating at −20°C for at least 1 h. Proteins were pelleted by centrifugation and air dried on ice for 15 min. Dried protein pellets were then resuspended in 100 μL of PBS. Protein suspensions were mixed with 500 μL of enzyme-linked immunosorbent assay (ELISA) coating buffer (50 mM Na_2_CO_3_, 500 mM NaHCO_3_ [pH 9.2]), and 150 μL of this mixture was added to the wells of 96-well microtiter plates. GFP was detected with MACS anti-GFP conjugated to horseradish peroxidase (HRP) (Miltenyi Biotec). Seventy-five microliters of tetramethyl benzidine (TMB; Thermo Scientific) HRP substrate was added to each well and incubated at room temperature with shaking for 30 min. Reactions were quenched with 75 μL of 2 N H_2_SO_4_, and the absorbance at 450 nm was measured using a Biotek Epoch 2 microplate reader.

Statistical significance (α = 0.05) was determined using Brown-Forsythe and Welch ANOVA tests and Dunnett’s T3 multiple comparisons.

### Quantitative reverse transcription-PCR (qRT-PCR).

To measure the levels of target transcripts, B. burgdorferi was cultured at 35°C, and 2-mL aliquots of bacteria were taken 1, 3, 5, 7, 9, and 11 days postinoculation. Cells were washed twice with PBS and frozen at −80°C. Frozen pellets were later resuspended in 60°C TRIzol (Thermo Fisher), and RNA was extracted using a Qiagen Mini RNA kit. Residual genomic DNA was depleted using on-column Turbo DNase (Thermo Fisher). The quality (RNA integrity number [RIN] > 7) and quantity of the RNA were determined using an Agilent 2100 Bioanalyzer and Agilent 6000 Nano chips. Purified RNA stocks were stored at −80°C.

cDNA for qPCR was produced using the iScript gDNA Clear cDNA synthesis kit (Bio-Rad). The cDNA stocks were diluted 1:100 into nuclease-free water for use as the template. Quantitative PCR was carried out using iTaq universal SYBR green Supermix (Bio-Rad) and a Bio-Rad CFX96 Touch real-time PCR thermocycler. Oligonucleotide primer sets for target genes were designed using the IDT Primer Quest Tool (https://www.idtdna.com/PrimerQuest/Home/Index). For all reactions, cycles involved melting at 95°C for 10 s, annealing at 55°C for 10 s, and extension at 72°C for 30 s. Each run was performed with technical triplicates of each reaction as well as no-template control reactions. Melting curves were performed with each run to validate the production of specific amplicons. Every reaction was tested with a reverse transcriptase-negative template to validate the absence of contaminating genomic DNA (gDNA). Quantification cycle (*C_q_*) values were normalized to *ftsK*, a gene whose expression is stable across different growth phases (55), using the Δ*C_q_* method, and fold difference was determined by the function 2^−Δ^*^Cq^*.

## References

[1] Kawakami H, Katayama T. 2010. DnaA, ORC, and Cdc6: similarity beyond the domains of life and diversity. Biochem Cell Biol 88:49–62. 10.1139/o09-154. 20130679

[2] McHenry CS. 2011. DNA replicases from a bacterial perspective. Annu Rev Biochem 80:403–436. 10.1146/annurev-biochem-061208-091655. 21675919

[3] Speck C, Messer W. 2001. Mechanism of origin unwinding: sequential binding of DnaA to double- and single-stranded DNA. EMBO J 20:1469–1476. 10.1093/emboj/20.6.1469. 11250912 PMC145534

[4] Kaur G, Vora MP, Czerwonka CA, Rozgaja TA, Grimwade JE, Leonard AC. 2014. Building the bacterial orisome: high-affinity DnaA recognition plays a role in setting the conformation of *oriC* DNA. Mol Microbiol 91:1148–1163. 10.1111/mmi.12525. 24443848 PMC3992943

[5] Ozaki S, Katayama T. 2012. Highly organized DnaA-*oriC* complexes recruit the single-stranded DNA for replication initiation. Nucleic Acids Res 40:1648–1665. 10.1093/nar/gkr832. 22053082 PMC3287180

[6] Dunham-Ems SM, Caimano MJ, Pal U, Wolgemuth CW, Eggers CH, Balic A, Radolf JD. 2009. Live imaging reveals a biphasic mode of dissemination of *Borrelia burgdorferi* within ticks. J Clin Invest 119:3652–3665. 10.1172/JCI39401. 19920352 PMC2786795

[7] Piesman J, Oliver JR, Sinsky RJ. 1990. Growth kinetics of the Lyme disease spirochete (*Borrelia burgdorferi*) in vector ticks (*Ixodes dammini*). Am J Trop Med Hyg 42:352–357. 10.4269/ajtmh.1990.42.352. 2331043

[8] De Silva AM, Fikrig E. 1995. Growth and migration of *Borrelia burgdorferi* in *Ixodes* ticks during blood feeding. Am J Trop Med Hyg 53:397–404. 10.4269/ajtmh.1995.53.397. 7485694

[9] Stevenson B, Seshu J. 2018. Regulation of gene and protein expression in the Lyme disease spirochete. Curr Top Microbiol Immunol 415:83–112. 10.1007/82_2017_49. 29064060

[10] Jutras BL, Verma A, Adams CA, Brissette CA, Burns LH, Whetstine CR, Bowman A, Chenail AM, Zuckert WR, Stevenson B. 2012. BpaB and EbfC DNA-binding proteins regulate production of the Lyme disease spirochete’s infection-associated Erp surface proteins. J Bacteriol 194:778–786. 10.1128/JB.06394-11. 22155777 PMC3272957

[11] Jutras BL, Chenail AM, Carroll DW, Miller MC, Zhu H, Bowman A, Stevenson B. 2013. Bpur, the Lyme disease spirochete’s PUR domain protein: identification as a transcriptional modulator and characterization of nucleic acid interactions. J Biol Chem 288:26220–26234. 10.1074/jbc.M113.491357. 23846702 PMC3764826

[12] Blinkova A, Hervas C, Stukenberg PT, Onrust R, O’Donnell ME, Walker JR. 1993. The *Escherichia coli* DNA polymerase III holoenzyme contains both products of the *dnaX* gene, tau and gamma, but only tau is essential. J Bacteriol 175:6018–6027. 10.1128/jb.175.18.6018-6027.1993. 8376347 PMC206684

[13] Dallmann HG, Thimmig RL, McHenry CS. 1995. DnaX complex of *Escherichia coli* DNA polymerase III holoenzyme. Central role of tau in initiation complex assembly and in determining the functional asymmetry of holoenzyme. J Biol Chem 270:29555–29562. 10.1074/jbc.270.49.29555. 7493998

[14] Cordeiro TFVB, Gontijo MTP, Jorge GP, Brocchi M. 2022. EbfC/YbaB: a widely distributed nucleoid-associated protein in prokaryotes. Microorganisms 10:1945. 10.3390/microorganisms10101945. 36296221 PMC9610160

[15] Tsuchihashi Z. 1991. Translational frameshifting in the *Escherichia coli dnaX* gene *in vitro*. Nucleic Acids Res 19:2457–2462. 10.1093/nar/19.9.2457. 1710356 PMC329457

[16] Tsuchihashi Z, Brown PO. 1992. Sequence requirements for efficient translational frameshifting in the *Escherichia coli dnaX* gene and the role of an unstable interaction between tRNA(Lys) and an AAG lysine codon. Genes Dev 6:511–519. 10.1101/gad.6.3.511. 1547945

[17] Larsen B, Gesteland RF, Atkins JF. 1997. Structural probing and mutagenic analysis of the stem-loop required for *Escherichia coli dnaX* ribosomal frameshifting: programmed efficiency of 50%. J Mol Biol 271:47–60. 10.1006/jmbi.1997.1162. 9300054 PMC7126992

[18] Messer W, Weigel C. 1997. DnaA initiator–also a transcription factor. Mol Microbiol 24:1–6. 10.1046/j.1365-2958.1997.3171678.x. 9140960

[19] Menikpurage IP, Woo K, Mera PE. 2021. Transcriptional activity of the bacterial replication initiator DnaA. Front Microbiol 12:662317. 10.3389/fmicb.2021.662317. 34140937 PMC8203912

[20] Jutras BL, Bowman A, Brissette CA, Adams CA, Verma A, Chenail AM, Stevenson B. 2012. EbfC (YbaB) is a new type of bacterial nucleoid-associated protein and a global regulator of gene expression in the Lyme disease spirochete. J Bacteriol 194:3395–3406. 10.1128/JB.00252-12. 22544270 PMC3434759

[21] Jutras BL, Chenail AM, Stevenson B. 2013. Changes in bacterial growth rate govern expression of the *Borrelia burgdorferi* OspC and Erp infection-associated surface proteins. J Bacteriol 195:757–764. 10.1128/JB.01956-12. 23222718 PMC3562092

[22] Solovyev V, Salamov A. 2011. Automatic annotation of microbial genomes and metagenomic sequences, p 61–78. *In* Li R (ed), Metagenomics and its applications in agriculture, biomedicine and environmental studies. Nova Science, Hauppauge, NY.

[23] Adams PP, Flores Avile C, Popitsch N, Bilusic I, Schroeder R, Lybecker M, Jewett MW. 2017. *In vivo* expression technology and 5’ end mapping of the *Borrelia burgdorferi* transcriptome identify novel RNAs expressed during mammalian infection. Nucleic Acids Res 45:775–792. 10.1093/nar/gkw1180. 27913725 PMC5314773

[24] Jutras BL, Savage CR, Arnold WK, Lethbridge KG, Carroll DW, Tilly K, Bestor A, Zhu H, Seshu J, Zuckert WR, Stewart PE, Rosa PA, Brissette CA, Stevenson B. 2019. The Lyme disease spirochete’s BpuR DNA/RNA-binding protein is differentially expressed during the mammal-tick infectious cycle, which affects translation of the SodA superoxide dismutase. Mol Microbiol 112:973–991. 10.1111/mmi.14336. 31240776 PMC6736767

[25] Speck C, Weigel C, Messer W. 1999. ATP- and ADP-*dnaA* protein, a molecular switch in gene regulation. EMBO J 18:6169–6176. 10.1093/emboj/18.21.6169. 10545126 PMC1171680

[26] Smith JL, Grossman AD. 2015. *In vitro* whole genome DNA binding analysis of the bacterial replication initiator and transcription factor DnaA. PLoS Genet 11:e1005258. 10.1371/journal.pgen.1005258. 26020636 PMC4447404

[27] Sekimizu K, Bramhill D, Kornberg A. 1987. ATP activates *dnaA* protein in initiating replication of plasmids bearing the origin of the *E. coli* chromosome. Cell 50:259–265. 10.1016/0092-8674(87)90221-2. 3036372

[28] Gille H, Messer W. 1991. Localized DNA melting and structural pertubations in the origin of replication, *oriC*, of *Escherichia coli in vitro* and *in vivo*. EMBO J 10:1579–1584. 10.1002/j.1460-2075.1991.tb07678.x. 2026151 PMC452823

[29] Riley SP, Bykowski T, Cooley AE, Burns LH, Babb K, Brissette CA, Bowman A, Rotondi M, Miller MC, DeMoll E, Lim K, Fried MG, Stevenson B. 2009. *Borrelia burgdorferi* EbfC defines a newly-identified, widespread family of bacterial DNA-binding proteins. Nucleic Acids Res 37:1973–1983. 10.1093/nar/gkp027. 19208644 PMC2665219

[30] Jutras BL, Jones GS, Verma A, Brown NA, Antonicello AD, Chenail AM, Stevenson B. 2013. Posttranscriptional self-regulation by the Lyme disease bacterium’s BpuR DNA/RNA-binding protein. J Bacteriol 195:4915–4923. 10.1128/JB.00819-13. 23974034 PMC3807498

[31] Picardeau M, Lobry JR, Hinnebusch BJ. 1999. Physical mapping of an origin of bidirectional replication at the centre of the *Borrelia burgdorferi* linear chromosome. Mol Microbiol 32:437–445. 10.1046/j.1365-2958.1999.01368.x. 10231498

[32] Ogura Y, Imai Y, Ogasawara N, Moriya S. 2001. Autoregulation of the *dnaA-dnaN* operon and effects of DnaA protein levels on replication initiation in *Bacillus subtilis*. J Bacteriol 183:3833–3841. 10.1128/JB.183.13.3833-3841.2001. 11395445 PMC95264

[33] Berenstein D, Olesen K, Speck C, Skovgaard O. 2002. Genetic organization of the *Vibrio harveyi* DnaA gene region and analysis of the function of the *V. harveyi* DnaA protein in *Escherichia coli*. J Bacteriol 184:2533–2538. 10.1128/JB.184.9.2533-2538.2002. 11948168 PMC134989

[34] Salazar L, Guerrero E, Casart Y, Turcios L, Bartoli F. 2003. Transcription analysis of the *dnaA* gene and *oriC* region of the chromosome of *Mycobacterium smegmatis* and *Mycobacterium bovis* BCG, and its regulation by the DnaA protein. Microbiology (Reading) 149:773–784. 10.1099/mic.0.25832-0. 12634345

[35] Jakimowicz D, Majka J, Lis B, Konopa G, Wegrzyn G, Messer W, Schrempf H, Zakrzewska-Czerwinska J. 2000. Structure and regulation of the *dnaA* promoter region in three *Streptomyces* species. Mol Gen Genet 262:1093–1102. 10.1007/pl00008652. 10660070

[36] Atlung T, Clausen E, Hansen FG. 1984. Autorepression of the *dnaA* gene of *Escherichia coli*. Adv Exp Med Biol 179:199–207. 10.1007/978-1-4684-8730-5_20. 6098152

[37] Flower AM, McHenry CS. 1986. The adjacent *dnaZ* and *dnaX* genes of *Escherichia coli* are contained within one continuous open reading frame. Nucleic Acids Res 14:8091–8101. 10.1093/nar/14.20.8091. 3534795 PMC311836

[38] Collier J. 2019. Cell division control in *Caulobacter crescentus*. Biochim Biophys Acta Gene Regul Mech 1862:685–690. 10.1016/j.bbagrm.2018.04.005. 29715525

[39] Hottes AK, Shapiro L, McAdams HH. 2005. DnaA coordinates replication initiation and cell cycle transcription in *Caulobacter crescentus*. Mol Microbiol 58:1340–1353. 10.1111/j.1365-2958.2005.04912.x. 16313620

[40] Keiler KC, Shapiro L. 2001. Conserved promoter motif is required for cell cycle timing of *dnaX* transcription in *Caulobacter*. J Bacteriol 183:4860–4865. 10.1128/JB.183.16.4860-4865.2001. 11466289 PMC99540

[41] Kato J, Katayama T. 2001. Hda, a novel DnaA-related protein, regulates the replication cycle in *Escherichia coli*. EMBO J 20:4253–4262. 10.1093/emboj/20.15.4253. 11483528 PMC149159

[42] Ishida T, Akimitsu N, Kashioka T, Hatano M, Kubota T, Ogata Y, Sekimizu K, Katayama T. 2004. DiaA, a novel DnaA-binding protein, ensures the timely initiation of *Escherichia coli* chromosome replication. J Biol Chem 279:45546–45555. 10.1074/jbc.M402762200. 15326179

[43] Zhang Q, Zhou A, Li S, Ni J, Tao J, Lu J, Wan B, Li S, Zhang J, Zhao S, Zhao GP, Shao F, Yao YF. 2016. Reversible lysine acetylation is involved in DNA replication initiation by regulating activities of initiator DnaA in *Escherichia coli*. Sci Rep 6:30837. 10.1038/srep30837. 27484197 PMC4971506

[44] Li S, Zhang Q, Xu Z, Yao YF. 2017. Acetylation of lysine 243 inhibits the *oriC* binding ability of DnaA in *Escherichia coli*. Front Microbiol 8:699. 10.3389/fmicb.2017.00699. 28473824 PMC5397419

[45] Carr KM, Kaguni JM. 1996. The A184V missense mutation of the *dnaA*5 and *dnaA*46 alleles confers a defect in ATP binding and thermolability in initiation of *Escherichia coli* DNA replication. Mol Microbiol 20:1307–1318. 10.1111/j.1365-2958.1996.tb02649.x. 8809781

[46] Old IG, Margarita D, Saint Girons I. 1993. Unique genetic arrangement in the *dnaA* region of the *Borrelia burgdorferi* linear chromosome: nucleotide sequence of the *dnaA* gene. FEMS Microbiol Lett 111:109–114. 10.1111/j.1574-6968.1993.tb06369.x. 8359672

[47] Fujikawa N, Kurumizaka H, Nureki O, Terada T, Shirouzu M, Katayama T, Yokoyama S. 2003. Structural basis of replication origin recognition by the DnaA protein. Nucleic Acids Res 31:2077–2086. 10.1093/nar/gkg309. 12682358 PMC153737

[48] Barbour AG. 1984. Isolation and cultivation of Lyme disease spirochetes. Yale J Biol Med 57:521–525. 6393604 PMC2589996

[49] Savage CR, Jutras BL, Bestor A, Tilly K, Rosa PA, Tourand Y, Stewart PE, Brissette CA, Stevenson B. 2018. *Borrelia burgdorferi* SpoVG DNA- and RNA-binding protein modulates the physiology of the Lyme disease spirochete. J Bacteriol 200:e00033-18. 10.1128/JB.00033-18. 29632088 PMC5971483

[50] Sutton MD, Kaguni JM. 1997. Threonine 435 of *Escherichia coli* DnaA protein confers sequence-specific DNA binding activity. J Biol Chem 272:23017–23024. 10.1074/jbc.272.37.23017. 9287298

[51] Zawilak-Pawlik AM, Kois A, Zakrzewska-Czerwinska J. 2006. A simplified method for purification of recombinant soluble DnaA proteins. Protein Expr Purif 48:126–133. 10.1016/j.pep.2006.01.010. 16517180

[52] Qi X, Sun Y, Xiong S. 2015. A single freeze-thawing cycle for highly efficient solubilization of inclusion body proteins and its refolding into bioactive form. Microb Cell Fact 14:24. 10.1186/s12934-015-0208-6. 25879903 PMC4343044

[53] Babb K, Bykowski T, Riley SP, Miller MC, Demoll E, Stevenson B. 2006. Borrelia burgdorferi EbfC, a novel, chromosomally encoded protein, binds specific DNA sequences adjacent to *erp* loci on the spirochete’s resident cp32 prophages. J Bacteriol 188:4331–4339. 10.1128/JB.00005-06. 16740939 PMC1482946

[54] Laniel MA, Béliveau A, Guérin SL. 2001. Electrophoretic mobility shift assays for the analysis of DNA-protein interactions. Methods Mol Biol 148:13–30. 10.1385/1-59259-208-2:013. 11357581

[55] Arnold WK, Savage CR, Brissette CA, Seshu J, Livny J, Stevenson B. 2016. RNA-Seq of *Borrelia burgdorferi* in multiple phases of growth reveals insights into the dynamics of gene expression, transcriptome architecture, and noncoding RNAs. PLoS One 11:e0164165. 10.1371/journal.pone.0164165. 27706236 PMC5051733

