# Supplementary material for: Borrelia burgdorferi DnaA and the Nucleoid-Associated Protein EbfC Coordinate Expression of the dnaX-ebfC Operon
Source: J Bacteriol. 2022 Dec 19;205(1):e00396-22. doi: 10.1128/jb.00396-22 (PMC9879097; doi:10.1128/jb.00396-22)
Supplement: Supplemental file 1 — Fig. S1 and S2 and Table S1. Download jb.00396-22-s0001.pdf, PDF file, 1.0 MB [file jb.00396-22-s0001.pdf]

Supplemental Material for:

Borrelia burgdorferi DnaA and the nucleoid-associated protein EbfC coordinate expression of the *dnaX-ebfC* operon.

Andrew C. Krusenstjerna[1], Timothy C. Saylor[1], William K. Arnold[1,†], Jamila S. Tucker[1], and Brian Stevenson[1,2]

1. Department of Microbiology, Immunology, and Molecular Genetics, University of Kentucky College of Medicine, Lexington, Kentucky, USA
2. Department of Entomology, University of Kentucky College of Agriculture, Food, and Ecology, Lexington, Kentucky, USA

Figure S1. Alignment of *dnaX* genes from different species of spirochetes to *E. coli*. Top: Alignment of slipped-strand region of *E. coli* (Ec) *dnaX* gene with those of *B. burgdorferi* (Bb) and the spirochetes *Treponema pallidum* (Tp) and *Leptospira interrogans* (Li). Bottom: Identities of the *dnaX* genes and DnaX proteins of *E. coli* (Ec), *B. burgdorferi* (Bb), *T. pallidum* (Tp), and *L. interrogans* (Li). The *dnaX* gene and protein sequences were taken from the type strains of *E. coli* (NC_000913), *B. burgdorferi* (NC_001318), *T. pallidum* (NC_000919), and *L. interrogans* (NZ_CP02414). The *E. coli dnaX* gene sequence was annotated to show the Shine-Dalgarno (RBS) sequence, slippery

sequence, and frameshift stop codon (16). Sequence alignments were performed on Geneious software using Clustal Omega.

Figure S2. Alignment of DnaA amino acid sequences from different bacterial species to *E. coli*. The DnaA protein sequences were taken from *E. coli* (NC_000913), *C. crescentus* (NC_002696), *B. subtilis* (NC_000964), *B. burgdorferi* (NC_001318), *T. pallidum* (NC_000919), and *T. denticola* (NC_002967). The sequences are annotated to show the Walker A motif (GxxxxGKT/S), Walker B motif (hhhhD), lysine (K) residues involved in acetylation, and the conserved alanine implicated in nucleotide binding. Sequence alignments were performed on Geneious software using Clustal Omega.

Table S1. Oligonucleotides used in these studies.

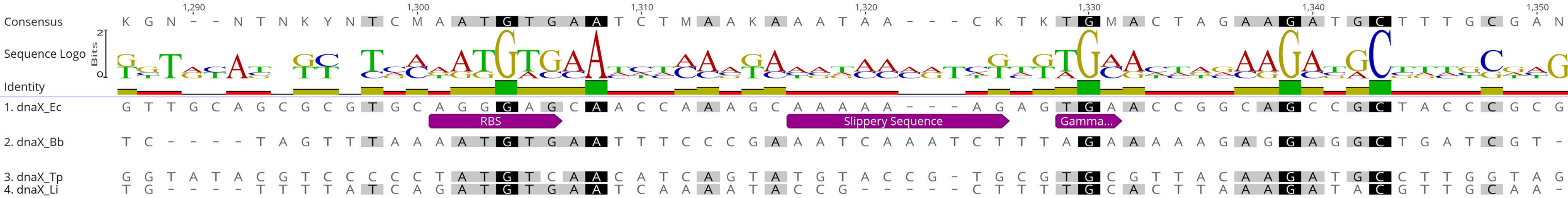

% Gene Identity

	dnaX Ec	*dnaX Bb*	*dnaX Tp*	*dnaX Li*
dnaX Ec	–	36.546%	36.947%	38.413%
dnaX Bb	36.546%	–	43.739%	46.619%
dnaX Tp	36.947%	43.739%	–	37.076%
dnaX Li	38.413%	46.619%	37.076%	–

% Protein Identity

	**DnaX *Ec***	**DnaX *Bb***	**DnaX *Tp***	**DnaX *Li***
**DnaX *Ec***	–	22.152%	22.946%	25.093%
**DnaX *Bb***	22.152%	–	31.933%	31.213%
**DnaX *Tp***	22.946%	31.933%	–	26.233%
**DnaX *Li***	25.093%	31.213%	26.233%	–

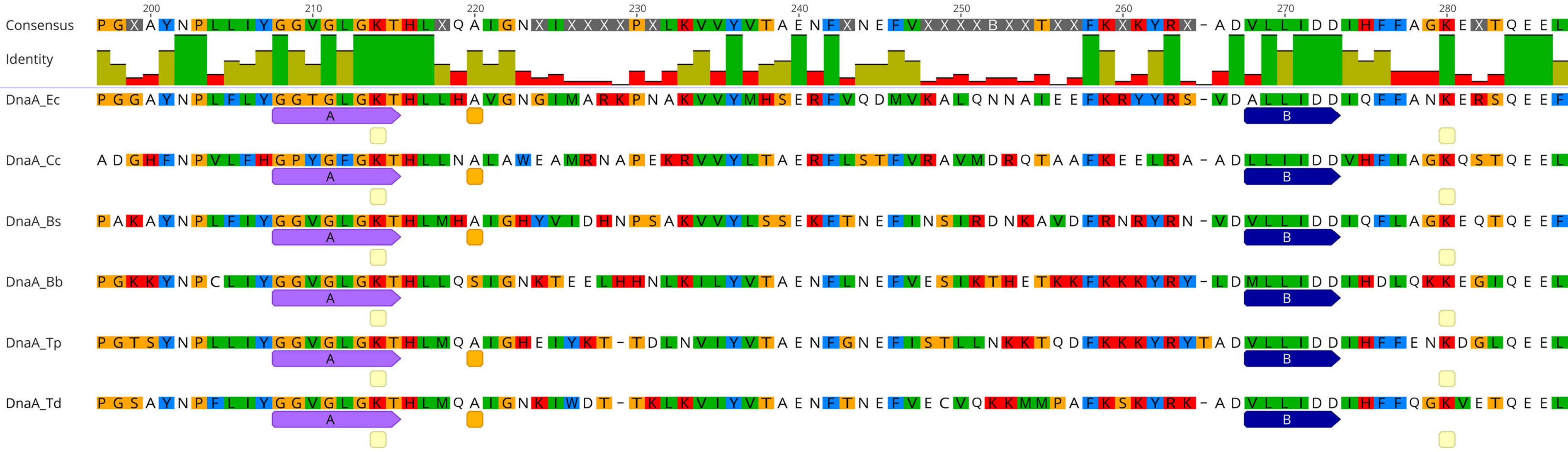

200 210 220 230 240 250 260 270 280
Consensus P G X A Y N P L L I Y G G V G L G K T H L X Q A I G N X I X X X P X L K V V Y V T A E N F X N E F V X X X B X X T X X F K X K Y R X - A D V L L I D D I H F F A G K E X T Q E E L
Identity
DnaA_Ec P G G A Y N P L F L Y G G T G L G K T H L L H A V G N G I M A R K P N A K V V Y M H S E R F V Q D M V K A L Q N N A I E E F K R Y Y R S - V D A L L I D D I Q F F A N K E R S Q E E F
A
B
DnaA_Cc A D G H F N P V L F H G P Y G F G K T H L L N A L A W E A M R N A P E K R V V Y L T A E R F L S T F V R A V M D R Q T A A F K E E L R A - A D L L I I D D V H F I A G K Q S T Q E E L
A
B
DnaA_Bs P A K A Y N P L F I Y G G V G L G K T H L M H A I G H Y V I D H N P S A K V V Y L S S E K F T N E F I N S I R D N K A V D F R N R Y R N - V D V L L I D D I Q F L A G K E Q T Q E E F
A
B
DnaA_Bb P G K K Y N P C L I Y G G V G L G K T H L L Q S I G N K T E E L H H N L K I L Y V T A E N F L N E F V E S I K T H E T K K F K K K Y R Y - L D M L L I D D I H D L Q K K E G I Q E E L
A
B
DnaA_Tp P G T S Y N P L L I Y G G V G L G K T H L M Q A I G H E I Y K T - T D L N V I Y V T A E N F G N E F I S T L L N K K T Q D F K K K Y R Y T A D V L L I D D I H F F E N K D G L Q E E L
A
B
DnaA_Td P G S A Y N P F L I Y G G V G L G K T H L M Q A I G N K I W D T - T K L K V I Y V T A E N F T N E F V E C V Q K K M M P A F K S K Y R K - A D V L L I D D I H F F Q G K V E T Q E E L
A
B

Table S1. Oligonucleotides used in this study.

Target	Name	Sequence	Purpose
dnaX-ebfC 5' UTR	*dnaX* F	GGTTTAATTTTGAACCCAA	EMSA probe 1, clone pJST01
	dnaX R*	CCTCCCTATGCTTTAATAAG	
dnaA promoter	P *dnaA* F	TAGCAACATAATTCATAAACCTTCCA	Clone pACK100
	P *dnaA* R	TTTGTCCTTGTTGGTTTTAGCACT	
oriC/*dnaN* promoter	*oriC* F	GATAGAGACAGAAATAATGACAAAG	Clone pACK101
	oriC R	GTTGTGTAGCATGATGCCTC	
dnaE promoter	P *dnaE* F	ATGGTAATTGAATTGGATAATCAAGGA	Clone pACK107
	P *dnaE* R	ACCTAGACCTAAAACTCATACCTAAA	
holA promoter	P *holA* F	GGATATATGTATCTGTAAATATTGC	Clone pACK111
	P *holA* R	CCAACAATAAATAAACCGC	
pCR2.1 backbone	M13 F	CAGGAAACAGCTATGAC	PCR TA probes
	M13 R*	GTAAAACGACGGCCAGT	
dnaA ORF	qPCR *dnaA* F	CATGTGACCGATCTCCTTCTG	qPCR
	qPCR *dnaA* R	CGACAATAGCTGCTCTGAGTT	
dnaX ORF	qPCR *dnaX* F	GTGGTAGCGTAAGAGATGCTTAT	qPCR
	qPCR *dnaX* R	GGTTAAGCCCATCTTGGATCT	
ebfC ORF	qPCR *ebfC* F	TGTGGTAAAGCAGGTAGCAATA	qPCR
	qPCR *ebfC* R	GAGACAGCATCATTTAAAGCAGAT	
dnaN ORF	qPCR *dnaN* F	GTCGGGAGAAGGTATGGTTAAA	qPCR
	qPCR *dnaN* R	CTCATCTGCTCCGTCATACAG	
dnaE ORF	qPCR *dnaE* F	CATTGGTTCTTGAGGGAATGAATAG	qPCR
	qPCR *dnaE* R	AGAGGCACATAGTCGGTTAAAG	
holA ORF	qPCR *holA* F	CAGCTGTAGGATTTGCTGAGA	qPCR
	qPCR *holA* R	GCTATATCTTCCTCCTCAATGGTATTA	
ftsK ORF	qPCR *ftsK* F	GACCTTCTGATGAGCCAATGT	qPCR
	qPCR *ftsk* R	GCTGCTCTGTTGTAACCTATCT	
Consensus EbfC motif	EbfC F*	GGATTTGTCTTTTTGTAACTTTCAATTTTA	EMSA probe 2
	EbfC R	TAAAATTGAAAGTTACAAAAAGACAAATCC	
Partial EbfC	DnaA F*	TCATAAAATTCTTTTAAAGTAATAGCTCTG	EMSA probe 3
	DnaA R	CAGAGCTATTACTTTAAAAGAATTTTATGA	
dnaX-ebfC 5' UTR	DPC1 F	TTAATTTTGAACCCAAATGCAATGTTTTGCTTTATGTTAAGATAT	*dnaX-ebfC* 5' UTR Competitor 1
	DPC1 R	ATATCTTAACATAAAGCAAAACATTGCATTTGGGTTCAAAATTAA	
dnaX-ebfC 5' UTR	DPC2 F	GGATTTGTCTTTTTGTAACTTTCAATTTTATTATATTTTTTATGT	*dnaX-ebfC* 5' UTR Competitor 2
	DPC2 R	ACATAAAAAATATAATAAAATTGAAAGTTACAAAAAGACAAATCC	
dnaX-ebfC 5' UTR	DPC3 F	TTCATAAAATTCTTTTAAAGTAATAGCTCTGAGATATAAATATGT	*dnaX-ebfC* 5' UTR Competitor 3
	DPC3 R	ACATATTTATATCTCAGAGCTATTACTTTAAAAGAATTTTATGAA	
dnaX-ebfC 5' UTR	DPC4 F	TGTATAGTATATATTTACTAATATTTTACTTATTAAAGCATAGGG	*dnaX-ebfC* 5' UTR Competitor 4
	DPC4 R	CCCTATGCTTTAATAAGTAAAATATTAGTAAATATATACTATACA	
dnaX-ebfC 5' UTR	DPC5 F	AAATGCAATGTTTTGCTTTATGTTAAGATATGGATTTGTCTTTTT	*dnaX-ebfC* 5' UTR Competitor 5
	DPC5 R	AAAAAGACAAATCCATATCTTAACATAAAGCAAAACATTGCATTT	
dnaX-ebfC 5' UTR	DPC6 F	TTTCAATTTTATTATATTTTTTATGTTTCATAAAATTCTTTTAAA	*dnaX-ebfC* 5' UTR Competitor 6
	DPC6 R	TTTAAAAGAATTTTATGAAACATAAAAAATATAATAAAATTGAAA	
dnaX-ebfC 5' UTR	DPC7 F	AGCTCTGAGATATAAATATGTTGTATAGTATATATTTACTAATAT	*dnaX-ebfC* 5' UTR Competitor 7
	DPC7 R	ATATTAGTAAATATATACTATACAACATATTTATATCTCAGAGCT	
Consensus EbfC motif	EbfC RNA**	GGAUUUGUCUUUUUGUAACUUUCAAUUUUA	EMSA RNA probe & competitor
flaB mRNA	*flaB* RNA	AGGCAAAAGGAUUUGCCAAAGUCAGAAAUU	EMSA RNA competitor

* Oligo for probe conjugated with an IRDye800 fluorescent tag.

** Oligo for probe conjugated with an Alexa 647 fluorescent tag.
